# The impact of previous cancer on overall survival of bladder cancer patients and the establishment of nomogram for overall survival prediction

**DOI:** 10.1097/MD.0000000000022191

**Published:** 2020-09-18

**Authors:** Zhengquan Wang, Yuan Zhou, Chao Guan, Yinman Ding, Sha Tao, Xiaoqi Huang, Liang Chen, Fei Zhang, Rentao Zhang

**Affiliations:** a Department of Urology Surgery, The People's Hospital of Xuancheng City, Xuanzhou, Xuancheng; b Department of Urology Surgery, The Second Affiliated Hospital of Bengbu Medical College, Longzi, Bengbu, China.

**Keywords:** bladder cancer, nomogram, second primary cancer, SEER, survival

## Abstract

To investigate the role of previous cancer on overall survival in patients with bladder cancer (BCa) and to establish an effective prognostic tool for individualized overall survival prediction.

A total of 78,660 patients diagnosed with BCa between 2000 and 2013 were selected from the Surveillance, Epidemiology, and End Results (SEER) database, among which 8915 patients had a history of other cancers. We compared the overall survival between patients with and without previous cancer after propensity score matching and we further established a nomogram for overall survival prediction.

Univariate and multivariate Cox analyses were used to determine independent prognostic factors. The calibration curve and concordance index (C-index) were used to assess the accuracy of the nomogram. Cox proportional hazards models and Kaplan–Meier analysis were used to compare survival outcomes.

BCa patients with previous cancer had worse overall survival compared with those without previous cancer (HR = 1.37; 95%CI = 1.32–1.42, *P* < .001). Cancers in lung prior to BCa had the most adverse impact on overall survival (HR = 2.35; 95%CI = 2.10–2.63; *P* < .001), and the minimal impact was located in prostate (HR = 1.16; 95%CI = 1.10–1.22; *P* < .001) for male and in gynecological (HR = 1.15; 95%CI = 1.02–1.30; *P* *=* .027) for female. The shorter interval time between 2 cancers and the higher stage of the previous cancer development, the higher risk of death. Age, race, sex, marital status, surgery, radiation, grade, stage, type of previous cancer as the independent prognostic factors were selected into the nomogram. The favorable calibration curve and C-index value (0.784, 95%CI = 0.782–0.786) indicated the nomogram could accurately predict the 1-, 3-, and 5-year overall survival rate of BCa patients.

Previous cancer has a negative impact on the overall survival of BCa patients and requires more effective clinical management. The nomogram provides accurate survival prediction for BCa patients and might be helpful for clinical treatment selection and follow-up strategy adjustment.

## 1 Introduction

Bladder cancer (BCa) is the sixth most common malignancy in the world.^[1]^ There are significant regional and gender differences in the incidence of BCa, with the highest incidence rate in Southern and Western Europe for males and in Northern America for females and with the lowest incidence rate in South Eastern and South Central Asia and sub-Saharan Africa for both sexes.^[2]^ Men are more likely than women to develop bladder cancer, at a rate of 3.5 to 1.^[2]^ Smoking is one of the most important risk factors for BCa and increases the recurrence of BCa, the risk is proportional to the intensity and duration of smoking.^[3–4]^ Another risk factor for BCa is occupational exposure to aromatic amines, polycyclic aromatic hydrocarbons and other industrial chemicals.^[5]^ With the popularization of urine cytology and cystoscopy, the early diagnosis rate of BCa has greatly improved, and approximately 75% to 85% of bladder cancers are limited to mucosa.^[6]^ Meanwhile, advances in treatment have improved survival in BCa patients, and the 5-year survival rate for BCa has reached approximately 77%.^[7]^

With significant improvements of early detection and treatment, the survival rate of cancer patients has greatly improved. The cancer death rate decreased by approximately 1.5% annually from 2005 to 2014.^[1]^ The number of cancer survivors in the United States was approximately 15.1 million in 2016, and the number could rise to 20.3 million within a decade.^[8]^ Cancer survivors are at a high risk of developing a second primary cancer (SPC), which significantly increases the risk of death among cancer survivors. According to data statistics, approximately 8% of cancer survivors may develop a SPC, and patients with 2 primary cancers have a worse prognosis than those with only 1.^[9–10]^ For cancer survivors, men are more than twice as likely as women to develop another type of primary cancer, while tobacco and alcohol may contribute to the increased risk of SPC.^[11]^ The impact of previous cancer on overall survival is inconclusive for patients with SPC. Previous cancer may not adversely affect the survival in patients with locally advanced lung cancer, but it do significantly adversely affect survival in patients with prostate cancer.^[12,13]^

Because of the large number of patients with BCa, there is a high risk that BCa will develop SPC. However, the effect of previous cancers on the survival of BCa patients is not fully understood. At the same time, individualized survival in patients with BCa as the SPC has not been studied. It is necessary to research the role of previous cancer on survival in BCa patients and to develop individualized survival predictions for the first time by population-based large-scale studies.

## 2 Methods

### 2.1 DATA acquisition

Data used for analysis were obtained from the largest cancer database in the United States, the Surveillance, Epidemiology and End Results (SEER) database. SEER database has 18 cancer registries covering about 28 percent of the cancer population in the United States.^[14]^ Extensive information on the SEER database of tumor patients has contributed significantly to the epidemiology of cancer.

### 2.2 Study population

This study selected patients diagnosed with BCa in the SEER database between 2000 and 2013. All cancer patients were confirmed by pathologically diagnosis. To minimize the pathological bias, we included only the most common pathological subtype of BCa, transitional cell carcinoma. Patients with missing information on race, sex, age, marital status, stage, grade, surgery, and radiation were excluded. BCa as second primary cancer was defined as when there was only one other type of cancer before BCa.

### 2.3 Statistical analysis

Kaplan–Meier curves were used to compare the effects of stage, type of previous cancer and time interval between cancers on survival. We used propensity score matching (PSM) to balance the clinical variables and reduce the statistical deviation. Information on race, sex, age, marital status, stage, grade, surgery, and radiation was propensity score matched between BCa patients with and without a previous cancer. Cox proportional hazards regression models were used for the multivariate analyses.

We further used univariate and multivariate Cox analyses to determine independent prognostic factors for the overall survival of BCa patients. The significant prognostic factors were used to develop a nomogram to predict the 1-, 3-, and 5-year overall survival rates. The accuracy of the prognostic nomogram was externally verified by the confidence interval (C-index) and calibration curves (bootstraps with 1000 re-samples). Generally, C-index value greater than 0.7 indicates that the predictive ability of the nomogram is satisfactory.^[15]^ In the calibration curves, the closer the prediction curve is to the observation curve, the more accurate the prognostic prediction is. All statistical analyses were performed by R software. A double-tailed *P* value <.05 was considered statistically significant.

### 2.4 Ethical approval

All data in this study were acquired from the SEER database with the purpose to research, thus the current study did not include any human participants or animals performed by any of the authors.

## 3 Results

A total of 78,660 patients diagnosed with BCa between 2000 and 2013 were screened from the SEER database, among which 8915 patients had a history of previous cancer. Patient information included age, race, sex, marital status, surgery, radiation, grade and stage, and all of which showed no difference in distribution between groups after PSM (*P* > .05 for all, Table 1). Previous cancers were classified by tissue system into prostate (46%), intestine (10%), skin (4%), urinary system (10%), lung (6%), gynecology (9%), hematologic and lymph (6%) and other types (9%), among which only 5% were distant-stage cancers. Approximately 70% of patients were developed a SPC within 5 years, and the average time interval between the previous cancer and BCa was approximately 4 years. In this study, all patients with BCa had the pathological type of transitional cell carcinoma, while only 25 cases of previous cancers (23 cases for kidney and 2 cases for ovary) had the pathological type of transitional carcinoma except renal pelvis and ureter.

**Table 1:**
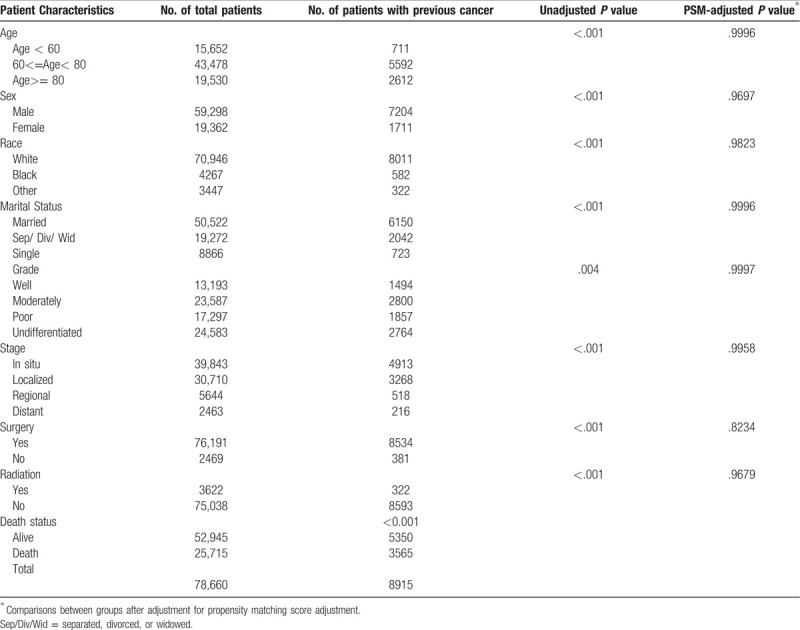
Baseline characteristics of bladder cancer patients included from SEER data cohort (N = 78,660) and between-group comparisons (No previous cancer vs previous cancer).

**Figure 1:**
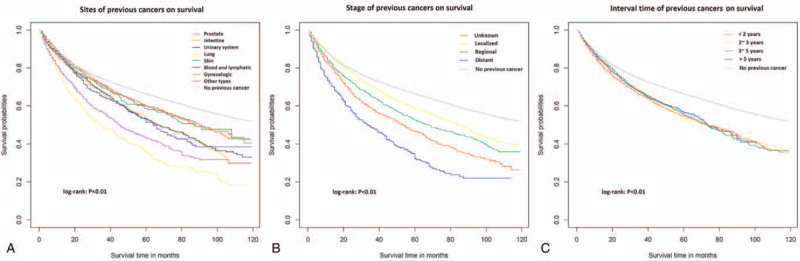
Survival comparison for sites (A), stages (B) and interval times (C) of previous cancers.

We made Kaplan–Meier curves to compare the effect of the stage, type and interval time of previous cancer on overall survival (Fig. 1). We used PSM to balance the clinical variables and reduce the statistical deviation, and found that patients with BCa had the worse overall survival compared with those without previous cancer (HR = 1.37; 95%CI = 1.32–1.42; *P* < .001). Lung cancer prior to BCa had the most adverse impact on overall survival (HR = 2.35; 95%CI = 2.10–2.63; *P* < .001), and the minimal impact was located in prostate (HR = 1.16; 95%CI = 1.10–1.22; *P* < .001) for male and in gynecological (HR = 1.15; 95%CI = 1.02–1.30; *P* *=* .027) for female. The shorter interval time between 2 cancers and the higher stage of the previous cancer development, the higher risk of death. (Table 2).

**Table 2:**
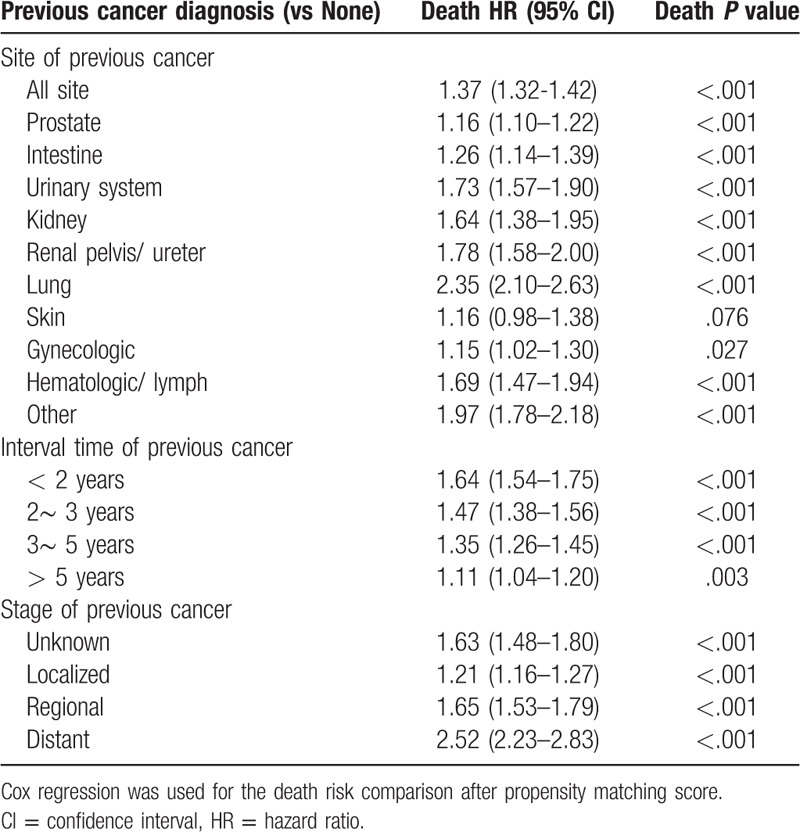
The comparison of death risk between bladder cancer patients with and without a previous cancer.

**Figure 2:**
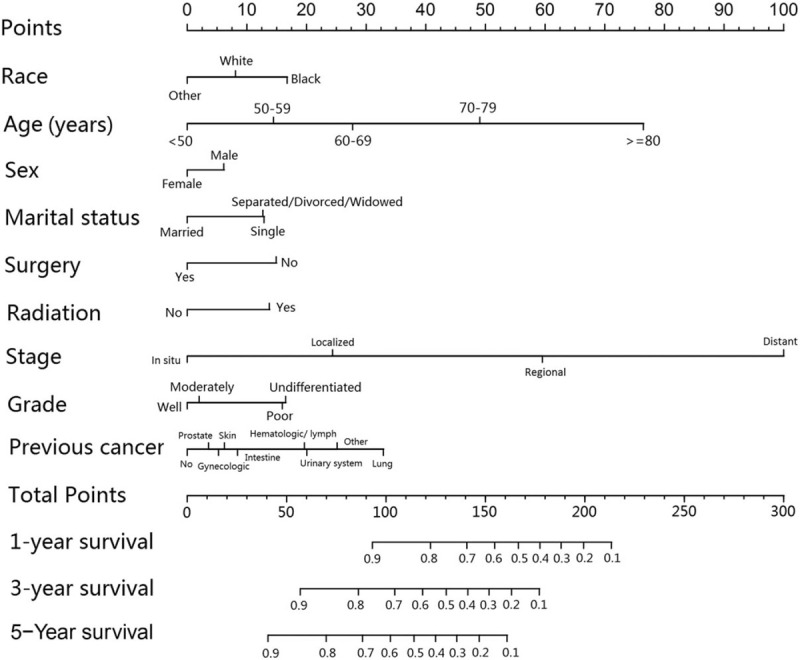
Survival nomogram for bladder cancer patients. (To use the nomogram, place the patients information on each variable axis, and draw a line to determine the points of the variable. The sum of the points is located on the total score axis. Next, draw a line down to the survival axis to determine 1-, 3-, and 5-year survival probability.).

**Figure 3:**
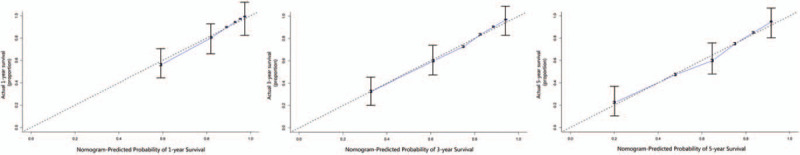
Calibration curves to predict 1-year (A), 3-year (B), and 5-year (C) survival in bladder cancer patients. The nomogram-predicted overall survival possibility is plotted on the X axial, and the actual overall survival possibility is plotted on the Y axial.

The results of the univariable and multivariable Cox regression analyses are listed in Table 3. Age, race, sex, marital status, surgery, radiation, grade, stage and previous cancer, as significant prognostic factors, were included in the nomogram (Fig. 2). Older age (compared with <50 years old, >  = 80 years old: HR = 7.12; 95%CI = 6.51–7.79; *P* < .001) and higher stage (compared with cancer in situ, distant: HR = 13.00; 95%CI = 12.30–13.73; *P* < .001) were associated with the most significant influence on overall survival in BCa patients (Table 3). The C-index value of the nomogram for prediction was 0.784 (95%CI = 0.782–0.786), and the calibration curve showed good agreement between the prediction and actual observation in the probability of 1-year, 3-year, and 5-year survival rates (Fig. 3). The favorable C-index value and calibration curve indicated that the prognostic nomogram could provide accurate 1-, 3-, and 5-year survival predictions for BCa patients.

**Table 3:**
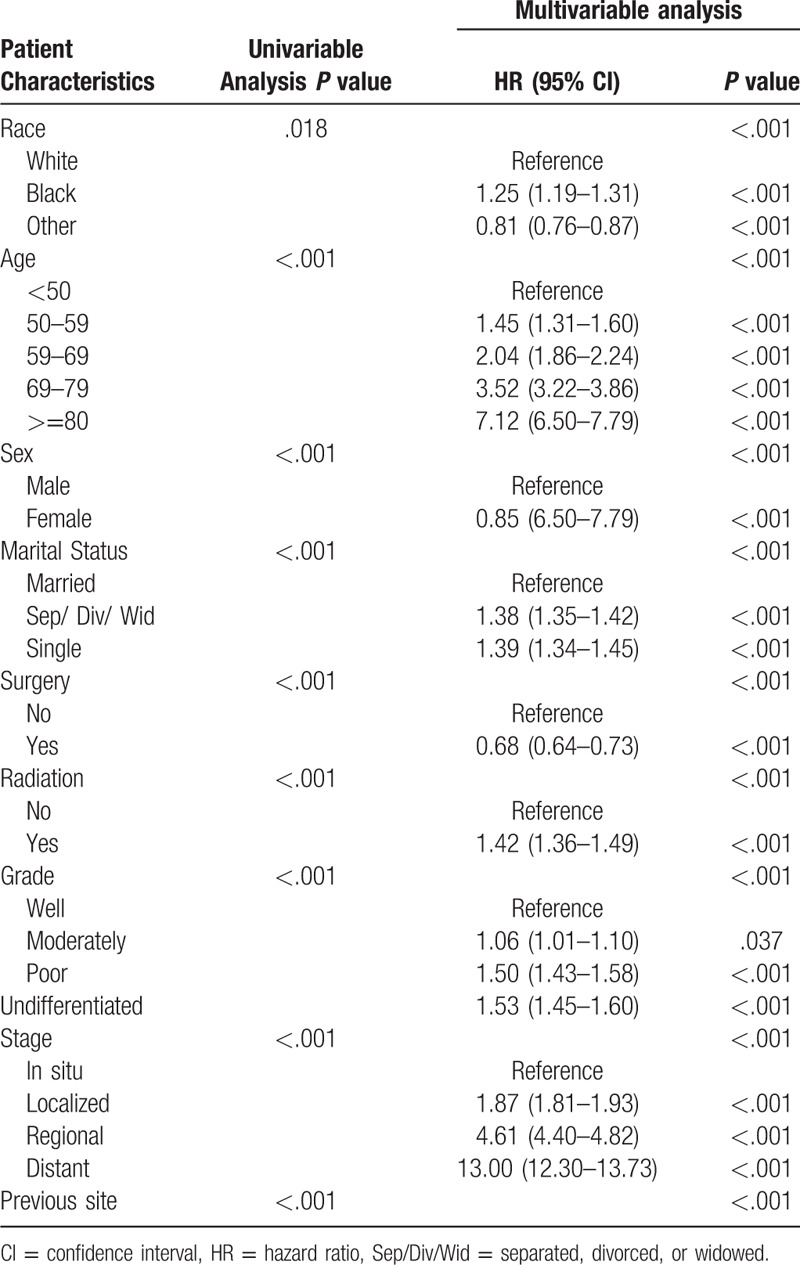
Univariate and multivariate analyses of overall survival in bladder cancer patients.

## 4 Discussion

Prostate cancer was found to be the most common cancer prior to BCa in this study, with a prevalence of 46% (n = 4072). In this study, the pathological types of bladder cancer were all transitional cell carcinoma, while prior prostate cancer was all non-transitional cell carcinoma. A study based on the SEER database reported that when prostate cancer as a SPC, prior BCa accounted for the largest proportion of all previous cancer types (28%), among which all prostate cancers were prostate adenocarcinoma, and only 1% of BCa was bladder adenocarcinoma.^[13]^ A long follow-up study reported that prostate cancer patients had an 18-fold higher risk of developing BCa, while BCa patients had a 19-fold higher risk of developing prostate cancer after age matched.^[16]^ BCa and prostate cancer had a high incidence in both directions, possibly due to the similar genetic backgrounds at the gene and protein levels (e.g., expression of p53 and pRb, overexpression of the UROC28 protein, and repair of N-acetyltransferase).^[17–19]^ Moreover, recurrent chronic inflammatory infections caused by factors such as urinary retention may also be a common risk factor for BCa and prostate cancer.^[20]^

Interestingly, when prostate cancer as the SPC, the previous cancer located in bladder had the most adverse impact on survival (HR = 5.00, 95%CI = 4.65–5.37, *P* < .001), followed by lung cancer (HR = 4.23, 95%CI = 3.64–4.91, *P* < .001).^[13]^ However, in this study, previous cancer located in the prostate had a minimal impact on survival for males when BCa was observed as the SPC. Why do changes in the sequence of prostate cancer and BCa affect survival so differently? We conjecture that the surgical modality may be one of the major influencing factors. The development of prostate cancer after BCa indicated that the prostate was not resected at the time of BCa development. However, when prostate cancer is followed by BCa, many patients may undergo radical prostatectomy when prostate cancer develops. Radical prostatectomy is the gold standard and first-choice treatment for prostate cancer within the located stage, and the surgical indications for surgery have gradually expanded.^[21]^ In the present study, there were 4072 BCa patients with a history of prostate cancer, among whom 3559 (87%) cases were within the located stage. We speculate that a large proportion of patients underwent radical prostatectomy when prostate cancer developed. It has previously been reported that patients with bladder transitional cell carcinoma are more prone to develop prostate infiltration, leading to prostate adenocarcinoma, and the prognosis is significantly worse than those of patients who undergo prostate resection.^[22]^ Lymphatic metastasis is more likely to occur when bladder transitional cell carcinoma invades the prostate, which seriously affects the prognosis of patients.^[23]^ However, detailed surgical information of patients is not available in the SEER database, we could not complete further statistical analysis to verify our conjecture, which we hope to confirm in future targeted studies.

Our study found that a history of previous cancer had a negative impact on overall survival in BCa patients, while previous cancer located in the lung had the most significant effect (compared with no previous cancer: HR = 2.35; 95%CI = 2.10–2.63; *P* < .001). Lung cancer is known to be particularly deadly and is the first leading cancer-related causes of death in the world.^[1]^ According to a study reported about SPCs, a diagnosis of lung cancer was the most lethal among patients with 2 malignancies, and lung cancer as the first primary cancer in all types of SPCs had the highest mortality rate.^[9]^ A prior history of cancer does not even affect overall survival in patients with locally advanced lung cancer.^[12]^ In the present study, patients with lung cancer prior to bladder cancer had the worst survival might not seem to be distinct.

The treatment of previous cancers did impact overall survival in BCa patients after PSM-adjusted (surgery: none vs yes, HR = 1.26, 95%CI = 1.17–1.36; *P* < .001; radiotherapy: yes vs. none, HR = 1.09, 95%CI = 0.82–1.43; *P* = .552; Tables not shown). However, due to the limited number of patients, we could not analyze the impact of treatment in each system of previous cancer on overall survival of BCa patients (e.g., only 15 cases not undergo surgery and 17 cases received radiotherapy in previous cancers of urinary system). Therefore, we chose the stage and interval time of the previous cancer for analysis and found that the shorter interval time between 2 cancers and the higher stage of the previous cancer development, the higher the risk of death.

Nomogram, a statistical tool, has been widely used in recent years by combining clinicopathological factors to individually predict survival in cancer patients.^[24–26]^ Therefore, we aim to develop a prognostic nomogram that can effectively predict the overall survival of patients with BCa and help clinicians in more effective individualized clinical management. As a result, a nomogram for 1-, 3-, and 5-year overall survival was established, and the C-index value of the nomogram for prediction and the calibration curve showed good agreement between the prediction and actual observation, which indicated that the proposed nomogram could accurately predict the 1-, 3, and 5-year overall survival rates of BCa patients. The prognostic nomogram could help clinicians evaluate the prognosis of BCa patients more intuitively and effectively and might be helpful in developing treatment strategies and adjusting follow-up plans. For example, for BCa patients with a poor prognosis, we could expand the treatment intensity and reduce the follow-up time to treat and monitor the disease more effectively.

Our study showed that race, age, sex, marital status, surgery, radiation, grade, stage, and previous cancer, as independent prognostic factors, could impact the survival of BCa patients. Stage had the most significant effect on survival in patients with BCa, followed by age. A previous study reported that black BCa patients had a higher risk of death than those from other races after age, sex, grade and stage matched, which may be related to economic income.^[27]^ Regarding the effect of gender on survival of BCa patients, it had been reported that males with BCa had a higher risk of death than female BCa patients, which may be related to smoking.^[3]^ A population-based study showed that the survival rate of married BCa patients in the United States was higher than that of single or divorced BCa patients.^[28]^ The effects of race, sex, and marital status on overall survival of BCa patients in our study were consistent with those previous reports, which could also prove the validity of our nomogram.

This study still has some limitations. First, different treatment regimens for the previous cancers may have affected the survival of patients. For example, chemotherapy may cause low tolerance or reduced therapeutic effects, and the missing treatment information may have introduced bias to our study. Second, due to the limited number of patients with previous cancers, we classified SPCs by the tissue system, and the survival rates of patients with different cancers in the same tissue system may be significant different. Although the average time interval between the previous cancer and BCa was approximately 4 years, there is still the possible that the 2 cancers actually developed in reverse order. Last, this study is retrospective in nature, and further prospective studies are needed to verify our results.

## 5 Conclusion

It is the first time to study the effect of previous cancer in BCa and establish an effective individualized overall survival prediction tool for BCa patients by a large-scale data set. We found a history of previous cancer has a negative impact on overall survival in BCa patients. This negative impact of previous cancer on overall survival was the most significant in lung cancer and had a minimal impact on survival in males with prostate cancer and in females with gynecological cancer. The shorter interval time between 2 cancers and the higher stage of the previous cancer development, the higher risk of death. The prognostic nomogram has been shown to accurate predict 1-, 3-, and 5-year overall survival rate in BCa patients. The proposed nomogram might be helpful to choose individualized clinical treatment and adjust follow-up strategies for BCa patients. BCa and prostate cancer patients may have a high incidence in both directions. When prostate cancer as a SPC, prior BCa had the most adverse impact on overall survival. However, prostate cancer prior to BCa had the minimal impact on overall survival for males. This may be related to whether the patient has undergone radical prostatectomy. Due to the limitations of this study, more targeted and further prospective studies are needed to validate our conjecture.

## Author contributions

**Conceptualization:** Rentao Zhang.

**Data curation:** Zhengquan Wang, Yuan Zhou.

**Formal analysis:** Zhengquan Wang, Chao Guan, Yinman Ding.

**Funding acquisition:** Chao Guan.

**Methodology:** Zhengquan Wang, Xiaoqi Huang.

**Supervision:** Yuan Zhou, Chao Guan.

**Validation:** Sha Tao, Liang Chen, Fei Zhang.

**Writing – original draft:** Zhengquan Wang, Yuan Zhou.

**Writing – review & editing:** Rentao Zhang.

## Abbreviations

Abbreviations: BCa = bladder cancer, CI = confidence interval, C-index = concordance index, HR = hazard ratio, PSM = propensity score matching, SEER = Surveillance Epidemiology and End Results, SPC = second primary cancer.
